# Association between Parathyroid Hormone Levels and Inflammatory Markers among US Adults

**DOI:** 10.1155/2014/709024

**Published:** 2014-03-23

**Authors:** Shih-Ping Cheng, Chien-Liang Liu, Tsang-Pai Liu, Yi-Chiung Hsu, Jie-Jen Lee

**Affiliations:** ^1^Department of Surgery, Mackay Memorial Hospital and Mackay Medical College, 92 Chung-Shan North Road, Section 2, Taipei 10449, Taiwan; ^2^Mackay Junior College of Medicine, Nursing and Management, 92 Shengjing Road, Taipei 11260, Taiwan; ^3^Department of Pharmacology and Graduate Institute of Medical Sciences, Taipei Medical University, 250 Wu-Hsing Street, Taipei 11031, Taiwan; ^4^Institute of Statistical Science, Academia Sinica, 128 Academia Road, Section 2, Nankang, Taipei 11529, Taiwan

## Abstract

*Background and Aims*. High levels of parathyroid hormone (PTH) appear to be associated with an increased mortality. Previous studies concerning the relationship of inflammatory markers with hyperparathyroidism have yielded inconsistent results. This study investigated whether serum PTH concentrations were independently associated with several inflammatory markers among the US adults. *Materials and Methods*. Using data from the National Health and Nutrition Examination Survey, we examined the relation between serum PTH and C-reactive protein (CRP), red cell distribution width (RDW), and platelet-to-lymphocyte ratio (PLR) levels with weighted linear regression. Additionally, we examined the relation with increased modified Glasgow Prognostic Score (mGPS) by using weighted logistic regression. *Results*. CRP, RDW, and PLR values increased with increasing serum PTH concentration. After extensively adjusting for covariates, CRP and RDW increased linearly and across PTH categories (all *P* < 0.001), while PLR marginally increased (*P* = 0.190 and *P* = 0.095 using PTH as a categorical and continuous variable, resp.). The odds ratio of increased mGPS was 1.11 and 1.31 across PTH categories and with increasing PTH levels continuously. *Conclusion*. These nationally representative data indicate that serum PTH levels are independently associated with several inflammatory markers in the US population. The casual relationship between PTH levels and inflammation remains to be elucidated.

## 1. Introduction

Parathyroid hormone (PTH) is the principle regulator of calcium and phosphorus homeostasis. PTH modulates osteoblast activity and osteoclast resorption, increases renal tubular reabsorption of calcium, and stimulates conversion of 25-hydroxyvitamin D to 1,25-dihydroxyvitamin D by 1*α*-hydroxylase in the kidney. In addition, PTH acts on bone cells to increase expression of fibroblast growth factor-23 (FGF-23) [1]. Higher PTH concentrations are associated with an increased mortality risk among general older populations [2, 3]. In the presence of hypovitaminosis D, absence of secondary hyperparathyroidism is characterized by lower rates of bone turnover and reduced mortality when compared with counterparts who manifest a physiological PTH elevation in response to vitamin D deficiency [4]. PTH has been considered to be a complementary biomarker in heart failure [5].

PTH stimulates interleukin-6 (IL-6) production by osteoblasts and liver cells [6, 7]. In turn, IL-6 may modulate acute-phase protein synthesis in the liver [8, 9]. It has been proposed to administrate vitamin D in the elderly to reduce serum levels of IL-6 and C-reactive protein (CRP) and, possibly, to decrease the risk of thromboembolic vascular events [10]. In this context, patients with hyperparathyroidism will, theoretically, have higher levels of IL-6, CRP, or tumor necrosis factor-*α* (TNF-*α*). However, previous studies have had conflicting results. Some showed elevated levels of these inflammatory markers in hyperparathyroidism [11–14], whereas others found that levels of CRP, IL-6, and leukocytes were similar in patients and controls [15, 16]. Furthermore, results for the effects of parathyroidectomy on subclinical inflammation were also inconsistent. Studies have showed decreased [11], increased [16–18], or unchanged [13, 19] levels of inflammatory markers after parathyroidectomy.

Such association studies may be underpowered to detect meaningful differences due to small sample sizes. Serum concentrations of 25-hydroxyvitamin D and PTH were found to independently associate with blood pressure and hypertension in the National Health and Nutrition Examination Survey (NHANES) in 2003 through 2006 [20]. The study did not specifically evaluate the relationship between PTH and CRP levels. In addition, other parameters such as IL-6 and TNF-*α* were not available in the NHANES database. Recently, we have validated the significance of another inflammatory marker, neutrophil-to-lymphocyte ratio, in differentiated thyroid cancer [21]. In the present study, we hypothesized that PTH levels might be associated with inflammatory status in the general US population. To test this hypothesis, we examined the cross-sectional associations between demographic, lifestyle, and serum factors and intact PTH using the large-scale data set released by the US NHANES 2003-2004 and 2005-2006.

## 2. Materials and Methods

### 2.1. Study Design and Population

NHANES provides nationally representative cross-sectional data for the health status of the US civilian noninstitutionalized population. In this analysis, we used data from the 2003-2004 and 2005-2006 NHANES that obtained serum PTH levels. The design and operation are available on the Centers for Disease Control and Prevention website [22, 23]. This analysis was limited to participants 20 years of age and older. Ethical approval for the study was obtained from the Research Ethics Review Board of National Center for Health Statistics, and all participants gave written informed consent.

### 2.2. Data Collection

Data were collected at all study sites by trained personnel using standardized procedures. Sociodemographic information such as age, gender, and race/ethnicity was recorded during the interview. Current smokers were defined as participants who were currently smoking and had smoked ≥100 cigarettes in their life. Blood pressure was measured at the mobile examination centers by physicians with mercury sphygmomanometers using a standard protocol. Up to four readings of systolic and diastolic blood pressure were averaged.

### 2.3. Serum Measurements

The complete blood count and leukocyte differential count were measured with the Beckman Coulter MAXM analyzer. Red cell distribution width (RDW) was derived from the coefficient of variation of the red cell volume distribution histogram. The platelet-to-lymphocyte ratio (PLR) was calculated. Serum creatinine concentration was measured by kinetic Jaffe assays with picrate (Beckman LX20). Estimated glomerular filtration rate (eGFR) was determined using the chronic kidney disease epidemiology collaboration (CKD-EPI) equation [24]. Serum total calcium was measured by an indirect ion-selective electrode method (Beckman LX20). When serum albumin level was <4.0 g/dL, serum calcium levels were corrected using the following formula: corrected calcium (mg/dL) = measured total calcium (mg/dL) + 0.8 ∗ [4.0 − serum albumin (g/dL)]. Serum 25-hydroxyvitamin D was measured using a Diasorin (formerly Incstar) 25(OH)D assay. Serum PTH was measured by electrochemiluminescence immunoassay on an Elecsys 1010 autoanalyzer (Roche Diagnostics). CRP was measured by latex-enhanced nephelometry on a Behring nephelometer.

The modified Glasgow Prognostic Score (mGPS) was calculated as previously described [25]. Participants with an elevated C-reactive protein concentration (>1 mg/dL) and a decreased albumin concentration (<3.5 g/dL) were assigned score 2. Those with an elevated C-reactive protein concentration (>1 mg/dL) were assigned score 1, and patients with a C-reactive protein concentration of ≤1 mg/dL and any albumin concentration were assigned score 0.

### 2.4. Statistical Analysis

All statistical analyses were computed by using survey commands of STATA (STATA Corporation) to incorporate sample weights and to adjust for clusters and strata of the complex sample design. Where distributions appeared nonnormal, we used natural log transformations to normalize their right-skewed distributions (white blood cell count, triglycerides, CRP, and PTH).

Serum PTH levels were stratified into clinically relevant categories (6–39, 40–59, 60–99, and ≥100). Potential confounding factors were chosen on the basis of previous studies or of their biologic plausibility. Tests of trend were calculated across PTH groups. Variance estimates were calculated using Taylor series linearization. Sample weights, which account for the differential probabilities of selection, nonresponse, and noncoverage, were incorporated into the variance estimation process. Univariate modeling was performed to determine whether a directional trend existed between variables and three inflammatory markers (CRP, RDW, and PLR). We examined whether the observed associations persisted within the subgroups stratified by eGFR and PTH. Subsequently, we constructed full multivariable linear regression models to test associations between inflammatory markers and serum PTH. Multivariate models were adjusted for age, sex, race/ethnicity, smoking status, glycohemoglobin, albumin, high-density lipoprotein, (logged) triglycerides, corrected total calcium, 25-hydroxyvitamin D, and eGFR. Trends across PTH categories were also assessed in linear regression models by using continuous (logged) PTH values. In addition, we performed logistic regression with a dichotomous outcome of increased mGPS (>0), adjusting simultaneously for the same covariates. All statistical tests were considered significant for *P* < 0.05.

## 3. Results

Amongst the 8948 participants who formed our main analysis sample, the weighted mean age was 49.4 years. The study sample consisted of 4322 men and 4626 women. Weighted mean PTH was 46.3 pg/mL (SE = 0.5). The characteristics of the study according to PTH levels are outlined in Table 1. The results indicate that with increasing PTH levels, age, body mass index, systolic and diastolic pressure, glycohemoglobin, and serum levels of creatinine tended to increase, while eGFR and serum levels of albumin, calcium, phosphorus, and vitamin D tended to decrease with increasing PTH levels. In all, eGFR level was a major determinant of PTH levels.

**Figure 1:**
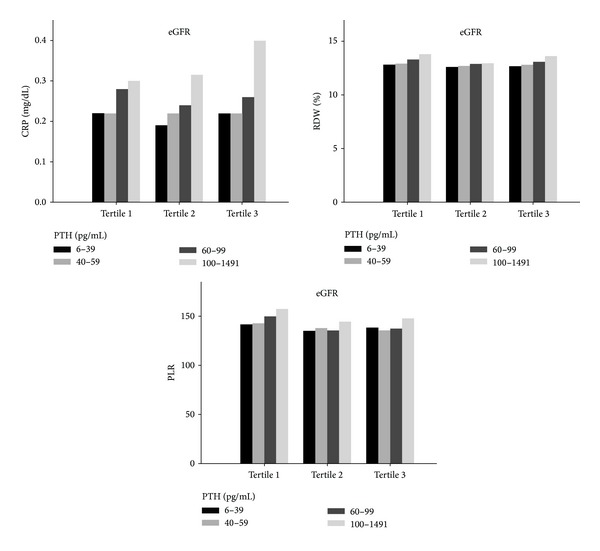
Unadjusted estimated glomerular filtration rate- (eGFR-) stratified levels of C-reactive protein (CRP), red blood cell distribution width (RDW), and platelet-to-lymphocyte ratio (PLR) by parathyroid hormone (PTH) categories.

Three inflammatory markers (CRP, RDW, and PLR) were examined in our study. The weighted mean (95% CI) CRP, RDW, and PLR was 0.49 (0.47–0.51) mg/dL, 12.87% (12.84%–12.91%), and 139.6 (137.7–141.5), respectively. The unadjusted eGFR-stratified levels of CRP, RDW, and PLR by PTH categories are displayed in Figure 1. CRP and RDW values increased at higher PTH categories independent of eGFR tertile (all *P* values <0.01). The unadjusted PLR tended to increase across PTH categories, but the statistically significant difference was observed only among participants in the lowest tertile of eGFR (*P* = 0.004).

Results of the multivariate linear regression predicting levels of three inflammatory markers are presented in Table 2. After adjusting for age and sex (Model 1), the linear relationships of PTH with CRP and RDW were statistically significant (*P* < 0.001). These relationships persisted after further adjusting for covariates including race/ethnicity, smoking status, glycohemoglobin levels (Model 2), and other potential confounders (Model 3). After further adjusting for PTH determinants including calcium, vitamin D, and eGFR (Model 4), CRP and RDW remained significantly associated with PTH categories (*P* < 0.001). Moreover, when (logged) PTH was entered as a continuous variable in linear regression models, it was independently associated with CRP and RDW (both *P* < 0.001). PLR also showed increasing trends. PLR was marginally associated with PTH after multivariate adjustment for covariables, assessed either by categories of or by continuous PTH (*P* = 0.190 and *P* = 0.095, resp.).

**Figure 2:**
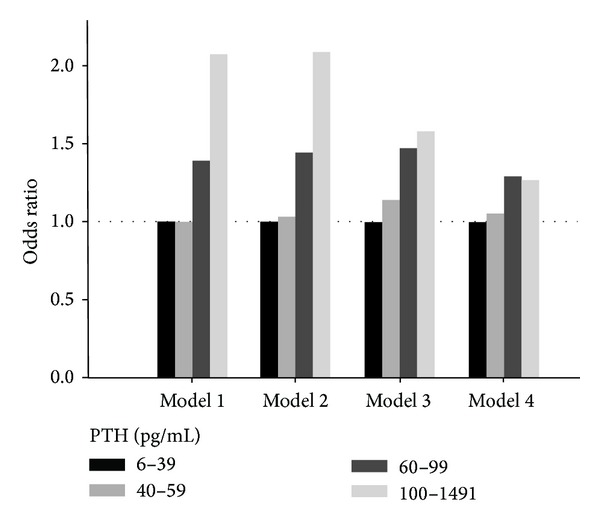
The odds ratios of increased modified Glasgow Prognostic Score according to parathyroid hormone (PTH) categories in NHANES 2003–2006. Model 1: adjusted for age and sex; Model 2: adjusted for age, sex, race/ethnicity, smoking status, and glycohemoglobin levels (continuous); Model 3: adjusted for variables in Model 2 plus albumin, triglycerides, and high-density lipoprotein levels (all continuous); Model 4: adjusted for variables in Model 3 plus corrected total calcium, 25-hydroxyvitamin D, and estimated glomerular filtration rate (all continuous).

Additionally, systemic inflammation was examined as a dichotomous variable (mGPS > 0). Excluding subjects with missing data of CRP (*n* = 2) and albumin (*n* = 34), 1083 (12%) participants had increased mGPS. We found significant correlations between mGPS and other inflammatory markers, namely, CRP, RDW, and PLR (all *P* < 0.001). Participants with increased mGPS had higher PTH concentrations (weighted mean 51.9 and 45.5, resp.; *P* < 0.001). As shown in Figure 2, the multivariate odds ratios for increased mGPS according to increasing PTH categories were 1, 1.05, 1.29, and 1.27 (odds ratio for trend 1.11). The trend remained significant when (logged) PTH levels were included in the logistic regression as a continuous variable (*P* values for Model 1 to Model 4 were <0.001, <0.001, <0.001, and 0.013, resp.). The odds ratio was 1.39, 1.42, 1.46, and 1.31 from Model 1 to Model 4, respectively. This is consistent with the observation that serum albumin levels were inversely associated with PTH concentrations.

## 4. Discussion

PTH is considered as a uremic toxin which may inflict damage on multiple organs [26]. Recently, we have shown that in dialysis patients with secondary hyperparathyroidism, the symptom burden had a negative impact on patients' quality of life, and parathyroidectomy significantly improved symptoms and quality of life [27]. Epidemiological studies have consistently reported higher total and cardiovascular mortality associated with PTH levels [2, 3, 28, 29]. In addition, associations with cancer mortality and noncancer, noncardiovascular mortality have been reported [2]. Pleiotropic effects of PTH are also reflected by numerous nonskeletal, nontraditional manifestations of primary hyperparathyroidism [30].

Accumulating evidence suggests that higher PTH levels may be associated low-grade inflammation. Adipose tissue from patients with primary hyperparathyroidism showed upregulation of inflammatory genes [31]. Dietary-induced hyperparathyroidism in rodents led to increased serum proinflammatory cytokine production [32]. Furthermore, in obese adolescents, PTH levels were closely correlated with CRP levels and triglycerides: high-density lipoprotein ratio [33]. The PTH level, but not the vitamin D level, is an independent predictor of metabolic syndrome [34]. However, a paradoxical increase in CRP levels after parathyroidectomy was observed in some studies [16, 18]. Therefore, the cause-and-effect relationship between PTH and inflammation remains unclear.

Inflammatory markers vary considerably across studies. The most extensively studied biomarkers of inflammation in cardiovascular disease are CRP and IL-6. The CRP level is consistently associated with the risk of cardiovascular disease [35]. Interestingly, elevated levels of CRP are associated with increased risk of several types of cancer [36]. In this study, we found a positive association between PTH and CRP, after adjustment for multiple potential confounders. Our results are in agreement with findings from epidemiological studies showing that PTH correlates with cardiovascular and even cancer mortality [2]. RDW is a quantitative measure of variability in the size of circulating erythrocytes, with higher values reflecting greater heterogeneity in cell sizes (i.e., anisocytosis). RDW is an easy, inexpensive, routinely reported parameter as a part of the complete blood count test. Recent studies exhibit a strong correlation between RDW and CRP [37–39]. Of note, data from NHANES III showed that RDW was associated with cardiovascular disease, cancer, and chronic lower respiratory tract disease [38]. RDW was shown to be a powerful predictor of mortality in community-dwelling older adults [40]. As a surrogate marker for the inflammatory state, we found that RDW levels strongly correlated with PTH and CRP levels. These findings further corroborate the association between PTH and inflammation.

Chronic inflammation is often associated with reactive thrombocytosis. Accompanied with systemic inflammation, release of various immunological mediators (including IL-6) increases circulating platelet counts as a result of megakaryocyte proliferation [41]. Platelets release the thromboxanes and other mediators, and consequently, patients with higher platelets may have increased inflammation. Smith and colleagues found that PLR is a prognostic marker in patients with periampullary cancer [42]. Subsequent study showed that PLR is an independent predictor of long-term mortality after non-ST segment elevation myocardial infarction [43]. In dialysis patients, PLR was positively correlated with IL-6 levels, and PLR was superior to neutrophil-to-lymphocyte ratio in terms of inflammation prediction [44]. Therefore, we used PLR as another inflammatory marker in the present study. Our analysis showed the trend of association between PLR and PTH levels. However, the association was no longer statistically significant in the fully adjusted model (i.e., Model 4). The nonsignificant results may be due to direct effects of PTH on platelets and lymphocytes [45–47]. Alternatively, there may be a nonlinear relationship between PTH levels and blood cell derangements induced by the acute phase reaction.

The combination of serum CRP and albumin has been used to derive the mGPS [25]. Hepatic albumin biosynthesis is downregulated by inflammatory stimuli as part of a negative acute phase reaction. Previous studies suggest that mGPS is superior to the original GPS and has greater consistency and is of more use [48]. The observation was based on the results that a low albumin concentration alone was uncommon and was not significantly associated with cancer-specific survival in many cancers. In this study, increased mGPS was analyzed as a dichotomous variable (>0 versus = 0) and taken as another marker of inflammation. Although mGPS is usually used to predict outcome in cancer patients, our results indicate that serum PTH levels were strongly associated with increased mGPS. Overall, our consistent findings buttress the validity of the association found between PTH levels and inflammation.

A major strength of our study is that it was based on nationwide, population-based sampling survey data. However, some limitations should be acknowledged. First, our analyses point to an association between PTH levels and inflammation but do not indicate a direction for the causal relationship because of the observational and cross-sectional study design. Second, other potential confounding variables could influence the findings. For example, in the NHANES database, there were no data on the FGF-23/Klotho axis. Higher FGF-23 levels were reported to be independently associated with higher levels of inflammatory markers in patients with chronic kidney disease [49]. Moreover, both systemic and local inflammation may decrease kidney Klotho expression, and Klotho downregulates inflammation [50]. Our finding may be a reflection of the association between low Klotho levels and inflammation. Third, no repeat measurements of PTH or other inflammatory markers were available, and therefore, the data set was limited to measures collected at a single time point for each participant. Finally, caution should be taken in interpreting results in relation to populations other than US adults.

## 5. Conclusion

In conclusion, using the NHANES data from the US adult population, we found an association between higher serum concentrations of PTH and several inflammatory markers (CRP, RDW, PLR, and mGPS). Studies have shown that vitamin D supplementation has a suppressive effect on PTH levels [51], but it is still unclear whether the suppression has any impact on clinical outcomes. Given that inflammation is involved in the pathophysiology of cardiovascular disease, cancer, aging, and other conditions, our results provide clues on which to base further investigations of the mechanistic aspects of the PTH-inflammatory link.

## Figures and Tables

**Table 1:** Characteristics according to parathyroid hormone (PTH) categories among the United States adults aged at least 20 years, NHANES 2003–2006.

	PTH group (pg/mL)				All
	6–39	40–59	60–99	100–1491	
*n*	4260	2952	1423	313	8948
Age (years)	44	52	56	63	49
Men (%)	49	48	47	47	48
Race (%)					
Mexican American	19	21	21	16	20
Other Hispanic	3	3	3	2	3
Non-Hispanic white	56	50	46	41	52
Non-Hispanic black	18	21	26	37	21
Other	4	4	3	4	4
Smoking (%)					
Current smoker	29	18	13	15	22
Former smoker	23	29	30	35	26
Never smoked	48	53	57	50	51
Body mass index (kg/m^2^)	27.6	29.0	30.4	31.0	28.6
Systolic blood pressure (mmHg)	120	126	132	137	125
Diastolic blood pressure (mmHg)	68	70	71	71	69
White blood cell count (/uL)	7589	7221	7209	7316	7398
Platelet count (1000/uL)	269	270	270	263	269
HbA1c (%)	5.5	5.6	5.7	5.8	5.6
Albumin (g/dL)	4.2	4.2	4.1	4.0	4.2
HDL (mg/dL)	56	54	55	53	55
LDL (mg/dL)	116	117	114	111	116
Triglycerides (mg/dL)	150	148	142	150	148
Creatinine (mg/dL)	0.9	0.9	1.0	1.6	0.9
eGFR (mL/min/1.73 m^2^)	98	90	83	63	92
Total calcium (mg/dL)	9.5	9.5	9.4	9.4	9.5
Corrected total calcium (mg/dL)	9.6	9.5	9.5	9.5	9.6
Phosphorus (mg/dL)	3.9	3.8	3.7	3.7	3.8
25(OH)D (ng/mL)	24.3	20.9	18.4	15.5	21.9

(1) Values are means incorporating sample weights and adjusted for clusters and strata of the complex sample design of NHANES 2003–2006.

(2) 25(OH)D: 25-hydroxyvitamin D; corrected total calcium: calcium corrected for serum albumin; eGFR: estimated glomerular filtration rate using the Chronic Kidney Disease Epidemiology Collaboration (CKD-EPI) equation; HbA1c: glycohemoglobin; HDL: high-density lipoprotein; LDL: low-density lipoprotein.

**Table 2:** Weighted mean values and the regression coefficients with standard errors (SEs) for C-reactive protein, red blood cell distribution width, and platelet-to-lymphocyte ratio by serum parathyroid hormone (PTH) levels among the United States adults aged at least 20 years, NHANES 2003–2006.

	Mean (SE)	Regression coefficient (SE)			
		Model 1	Model 2	Model 3	Model 4
C-reactive protein (mg/dL)					
PTH group (pg/mL)					
6–39	0.47 (0.01)	0	0	0	0
40–59	0.47 (0.02)	0.021 (0.044)	0.041 (0.044)	0.089 (0.040)	0.088 (0.038)
60–99	0.55 (0.02)	0.162 (0.032)	0.195 (0.034)	0.189 (0.035)	0.183 (0.037)
100–1491	0.75 (0.07)	0.339 (0.068)	0.363 (0.066)	0.194 (0.065)	0.175 (0.068)
*P* value for trend		<0.001	<0.001	<0.001	<0.001
Continuous PTH		0.085 (0.031)	0.109 (0.031)	0.139 (0.029)	0.138 (0.031)
*P* value		0.011	0.001	<0.001	<0.001
Red blood cell distribution width (%)					
PTH group (pg/mL)					
6–39	12.72 (0.02)	0	0	0	0
40–59	12.87 (0.02)	0.069 (0.022)	0.087 (0.022)	0.106 (0.020)	0.057 (0.022)
60–99	13.16 (0.04)	0.320 (0.039)	0.337 (0.040)	0.332 (0.038)	0.239 (0.040)
100–1491	13.68 (0.08)	0.770 (0.085)	0.757 (0.087)	0.667 (0.086)	0.504 (0.084)
*P* value for trend		<0.001	<0.001	<0.001	<0.001
Continuous PTH		0.299 (0.032)	0.315 (0.032)	0.322 (0.032)	0.231 (0.031)
*P* value		<0.001	<0.001	<0.001	<0.001
Platelet-to-lymphocyte ratio					
PTH group (pg/mL)					
6–39	138.0 (1.2)	0	0	0	0
40–59	139.0 (1.1)	−0.752 (1.171)	−1.674 (1.226)	−1.007 (1.253)	−0.480 (1.324)
60–99	142.6 (1.0)	1.778 (1.200)	0.399 (1.238)	0.713 (1.336)	1.059 (1.565)
100–1491	153.9 (3.9)	11.7462 (3.603)	9.641 (3.542)	9.353 (3.770)	7.454 (4.271)
*P* value for trend		0.004	0.114	0.085	0.190
Continuous PTH		3.183 (0.962)	1.679 (1.030)	2.193 (1.086)	2.444 (1.416)
*P* value		0.002	0.114	0.052	0.095

Linear regression analyses were conducted using serum PTH as both categorical and continuous variable. Model 1: adjusted for age and sex; Model 2: adjusted for age, sex, race/ethnicity, smoking status, and glycohemoglobin levels (continuous); Model 3: adjusted for variables in Model 2 plus albumin, triglycerides, and high-density lipoprotein levels (all continuous); Model 4: adjusted for variables in Model 3 plus corrected total calcium, 25-hydroxyvitamin D, and estimated glomerular filtration rate (all continuous).
